# Unifying cardiovascular modelling with deep reinforcement learning for uncertainty aware control of sepsis treatment

**DOI:** 10.1371/journal.pdig.0000012

**Published:** 2022-02-17

**Authors:** Thesath Nanayakkara, Gilles Clermont, Christopher James Langmead, David Swigon

**Affiliations:** 1 Department of Mathematics, University of Pittsburgh, Pittsburgh, PA, 15213, United States of America; 2 Department of Critical Care Medicine, The Clinical Research, Investigation, and Systems Modeling of Acute Illness (CRISMA) Center, University of Pittsburgh School of Medicine Pittsburgh, PA, 15213, United States of America; 3 Computational Biology Department, School of Computer Science, Carnegie Mellon University, Pittsburgh, PA, 15213, United States of America; 4 Department of Mathematics, McGowan Institute for Regenerative Medicine, University of Pittsburgh, Pittsburgh, PA, 15213, United States of America; National University of Singapore, SINGAPORE

## Abstract

Sepsis is a potentially life-threatening inflammatory response to infection or severe tissue damage. It has a highly variable clinical course, requiring constant monitoring of the patient’s state to guide the management of intravenous fluids and vasopressors, among other interventions. Despite decades of research, there’s still debate among experts on optimal treatment. Here, we combine for the first time, distributional deep reinforcement learning with mechanistic physiological models to find personalized sepsis treatment strategies. Our method handles partial observability by leveraging known cardiovascular physiology, introducing a novel physiology-driven recurrent autoencoder, and quantifies the uncertainty of its own results. Moreover, we introduce a framework for uncertainty-aware decision support with humans in the loop. We show that our method learns physiologically explainable, robust policies, that are consistent with clinical knowledge. Further our method consistently identifies high-risk states that lead to death, which could *potentially* benefit from more frequent vasopressor administration, providing valuable guidance for future research.

Sepsis is a major host response to infection which can result in tissue damage, organ damage and death. The mortality and economic burden of sepsis is very large. In the U.S., sepsis is responsible for 6% of all hospitalizations and 35% of all in-hospital deaths [1, 2], and an economic burden of more than $20B per year [3]. The treatment of sepsis is extremely challenging, due to the high variability among patients, with respect to both the progression of the disease, the host response to infection, and the response to medical interventions, suggesting the need for a dynamic and personalized approach to treatment [4–6]. Presently, the search for treatment strategies to optimize sepsis patient outcomes remains an open challenge in critical care medicine, despite decades of research.

Recently, there has been considerable interest in the application of Reinforcement Learning (RL) [7] to extract vasopressor and intravenous (IV) fluid treatment policies (i.e., strategies) for septic patients from electronic health records data (ex. [8–12]). Informally, the goal is to learn a policy that maps the patient’s current state to an action (i.e., medical intervention), so as to maximize the chances of future recovery. The RL framework is well-matched to the actual behaviors of physicians, who continuously observe, interpret, and react to their patient’s condition. The promise of RL in medicine is that we *might* be able to find policies that outperform humans (as it has in other domains, ex. [13–15]), by automatically personalizing the treatment strategy for each patient, as opposed to using one that is expected to work well on the *typical* patient [16, 17]. However, there are many challenges that must be met before RL can be used to guide medical decision making in real-life settings [18].

A particularly severe challenge is partial observability of patient state. Despite the richness of data collected at the ICU, the mapping between true patient states and clinical observables is often ambiguous. We believe that this ambiguity can be reduced through the use of mechanistic mathematical models of physiology that relate observables to a more complete representation of the patient’s cardiovascular state. Such models are plentiful in the literature, and embody decades of research in physiology and medicine. Our proposed solution integrates, for the first time, a clinically relevant mechanistic model into a Deep RL framework. The specific model we use was chosen because it estimates the unobservable aspects of cardiovascular state that are relevant to specific interventions (vasopressors and IV fluids), and the clinician’s goals—counteracting hypovolemia, vasodilation, and other physiological disturbances. This model is integrated into our framework using a self-trained deep recurrent autoencoder that uses a variety of inputs, including the patient’s vital signs, organ function scores, and previous treatments.

The second challenge addressed by our framework is uncertainty in the learned policy, and thus the expected outcomes. Similar to previous efforts to extract sepsis treatment policies from retrospective data (ex. [8]), our method works in the Batch Reinforcement Learning setting [19], where the agent cannot explore the environment freely. In this setting, it is well known that RL can perform poorly [20], if the agent encounters states that are rare or even unobserved in the training data. For this reason, it has been argued that all forms of uncertainty should be quantified in any application of Artificial Intelligence to Medicine [21]. Thus, we quantify model uncertainty (This should not be confused with the model-based vs model-free RL distinction, because once we have inferred latent states, our approach qualifies as ‘model-free’. The literature also uses the term epistemic uncertainty and parametric uncertainty for model uncertainty.) via bootstrapping and take a distributional approach to factor in environment uncertainty. We also propose a decision framework where the clinician is presented with a quantitative assessment of the distribution over outcomes for each state-action pair.

## 1 Background and related work

### 1.1 Reinforcement learning

Reinforcement Learning is a framework for optimizing sequential decision making. In its standard form, a Markov Decision Process (MDP), consisting of a 5-tuple (*S*,*A*,*r*,*γ*,*p*) is the framework considered. Here, *S* and *A* are state and action spaces, r:(S,A,S)→R is a reward function, *p* : (*S*, *A*, *S*) → [0, ∞) denotes the unknown environment dynamics, which specifies the distribution of the next state *s*′, given the state-action pair (*s*, *a*), and *γ* is a discount rate applied to rewards. A policy is (a possibly stochastic) mapping from *S* to *A*. The agent aims to compute the policy *π* which maximizes the expected future reward *E*_*p*,*π*_[Σ_*t*_*γ*^*t*^*r*_*t*_]. In the partially observed setting there is a distinction between the observations, denoted as *o*_*t*_, and the state *s*_*t*_, and the environment dynamics includes the conditional probability density *p*(*o*_*t*_|*s*_*t*_). This extends the MDP formalism to that of Partially Observed Markov Decision Process (POMDP).

The search for of an optimal policy can be performed in several ways, including the iterative calculation of the *value function*, $V^{\pi }\left(s\right)=E_{p,\pi }\left[\Sigma_{t}\gamma^{t}r_{t}\left(s_{t},a_{t}\right)|s_{0}=s,\pi \right],\forall s\in S$, which returns the expected future discounted rewards when following policy *π* and starting from the state *s*, or the *Q-function*, $Q^{\pi }\left(s,a\right)=E_{p,\pi }\left[\Sigma_{t}\gamma^{t}r_{t}\left(s_{t},a_{t}\right)|s_{0}=s,\pi ,a_{0}=a\right],\forall s\in S,a\in A$, which returns the expected future reward when choosing action *a* in state *s*, and then following policy *π*. Central to many RL algorithms is the Bellman equation [22]:

$$\begin{array}{c} Q^{\pi }\left(s,a\right)=E_{p}\left[r\left(s,a\right)\right]+\gamma E_{p,\pi }\left[Q^{\pi }\left(s^{\prime },a^{\prime }\right)\right], \end{array}$$ (1)

and the Bellman optimality equation:

$$\begin{array}{c} Q^{*}\left(s,a\right)=E_{p}\left[r\left(s,a\right)\right]+\gamma E_{p}\left[\underset{a^{\prime }\in A}{\text{max}}\;Q^{*}\left(s^{\prime },a^{\prime }\right)\right] \end{array}$$ (2)

(where Q*(*s*, *a*) is the optimal *Q* function, and *s*′ denotes the random next state).

### 1.2 Distributional and uncertainty aware reinforcement learning

Distributional Reinforcement Learning [23–25] extends traditional RL methods by estimating the entire return distribution from a given state, rather than simply an expected value. It has been shown that distributional RL can achieve superior performance in the context of Batch RL [26]. For this reason, and because distributions are relevant to our overall goal of providing clinicians with an assessment of the range of possible outcomes for each state-action pair, we employ Categorical Distributional RL [23]. Here the state, action value distribution is approximated by a discrete distribution with equally spaced support. Further, we employ Deep Ensembles [27] to quantify the uncertainty associated with each state action pair. These ensembles are constructed using bootstrap estimates, as explained in the methods section.

### 1.3 Reinforcement learning in medicine

Reinforcement Learning has been used for various healthcare applications. References [17] and [16] provide comprehensive surveys of healthcare and critical care applications respectively. In the specific context of sepsis treatment, Komorowski *et al*. [8] used a discrete state representation created by clustering patient physiological readouts, and a 25 dimensional discrete action space to compute optimal treatment strategies using dynamic programming based methods. Others have considered continuous state representations [9] and partial observability [10].

**Fig 1:**
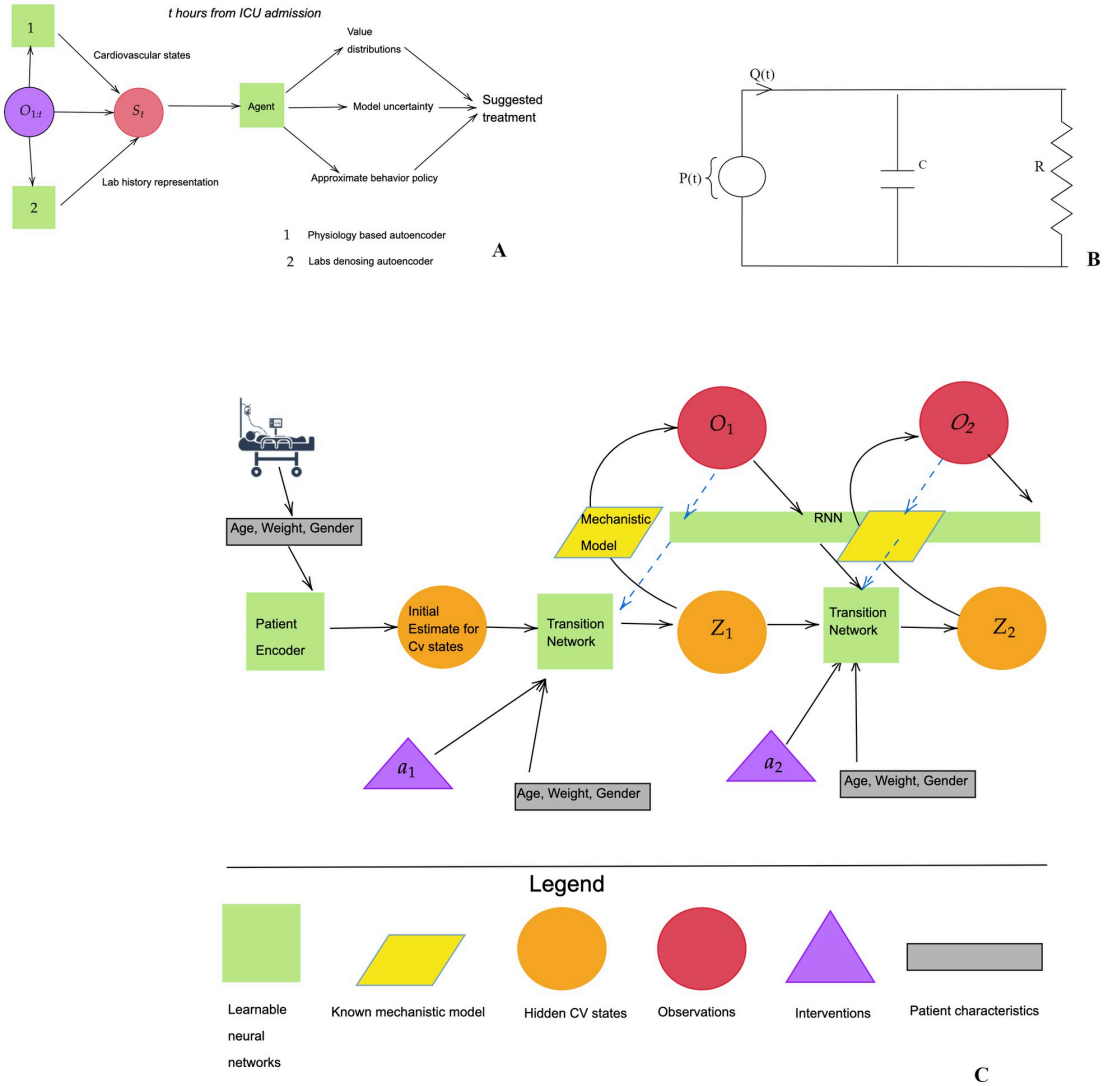
**Proposed decision support system (A)**: We use the compete patient history, which includes, vitals, scores, and labs, and previous treatment, to infer hidden states. These would all combine to make the state *S*_*t*_. Our trained agent, takes this state and outputs value distributions for each treatment, its own uncertainty, and an approximate clinician’s policy. We then factor in all 3 to propose uncertainty-aware treatment strategies. **The electrical analog of the cardiovascular model (B)** This provides a lumped representation of the resistive and elastic properties of the entire arterial circulation using just two elements, a resistance R and a capacitance C. This model is used to derive algebraic equations relating R, C, stroke volume (SV), filling time (T), to heart rate (F) and pressure. The Cardiac Output (CO) can be then computed as (SV)F. These equations define the decoder of the physiology-driven autoencoder. **Complete physiology-driven autoencoder network structure (C)** Patient history is sequentially encoded using three neural networks. A patient encoder computes initial cardiovascular state estimates using patient characteristics, a recurrent neural network (RNN) encodes the past history of vitals and scores, up to and including the current time point, and a transition network which takes the previous cardiovascular state, the action and the history representation to output new cardiovascular state estimates.

Our proposed decision support system is based on a preference score as shown in Fig 1A. In contrast to previous work, we choose a lower dimensional action space (9 actions), to ensure sufficient coverage in the training data, and a reduced decision time-scale, to be more aligned with clinical practice. The short time scale also provides a clinical justification for the less granular action space. Our rewards are based on previous work [9] (see Methods), which has intermediate SOFA-based rewards, and ±15 terminal rewards, depending on survival.

## 2 Results

### 2.1 Trajectory reconstruction using a physiology-driven autoencoder

**Fig 2:**
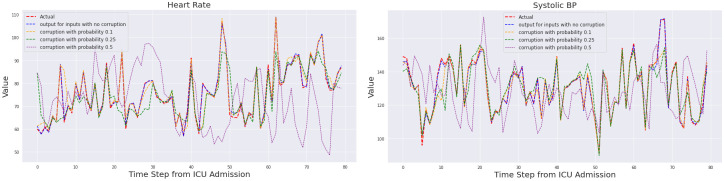
Reconstruction of two validation patient trajectories using different levels of corruption using the physiology-driven autoencoder, **Left**: Heart Rate. **Right**: Systolic Blood Pressure.

One of the key features of our method is the physiology-driven structure of the autoencoder that represents the cardiovascular state of the patient (see Fig 1B and 1C). The decoder of this autoencoder is a set of algebraic equations that map the latent state to observable, and clinically relevant physiological parameters, such as heart rate and blood pressure. Fig 2 shows selected reconstructed trajectories for one representative patient, using various levels of data corruption (see Methods). As the figure illustrates, the model successfully reconstructs the observable outputs and their trends with corruption probabilities as high as 25%. It is only at extreme levels of corruption (50%) that the model’s accuracy degrades. Such robustness to moderate levels of corruption was typical among training and validation patient trajectories. We thus conclude that the autoencoder has learned an effective representation of the cardiovascular state of the patient.

Below, we present (in Table 1) average unnormalized mean square error of the four dimensional output, per time step to the nearest integer.

**Table 1:** Mean square error of reconstruction.

Corruption probability	MSE per time step
0%	6
10%	45
25%	59
50%	258

### 2.2 Value distributions and expected values

**Fig 3:**
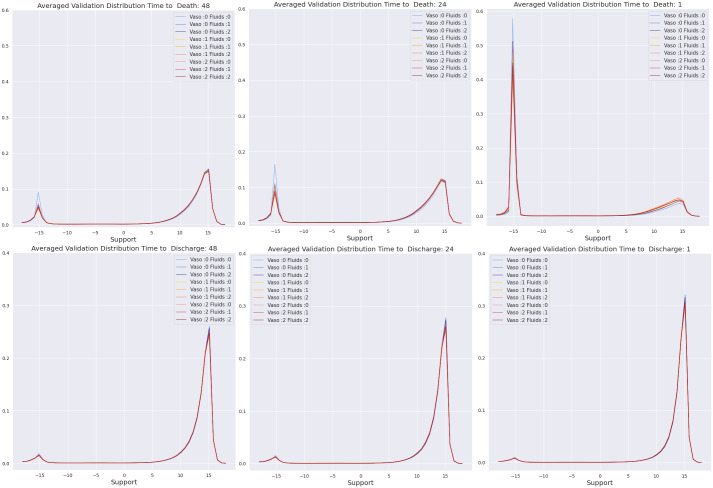
Value distributions for validation patients averaged according to different times from death or discharge, **Top row**: Non Survivors. **Bottom row**: Survivors.

We next investigated whether the learned values are generalizable, consistent with clinical knowledge, and correlated with the risk of death in non-survivors. To do this, we examined the value distributions that are produced at each time-step for patients in the validation set, stratified by outcome (i.e., survivor vs non-survivor). Fig 3 plots the average value distributions output for non-survivors (top) and survivors (bottom) at 48, 24, and 1 hour from death or discharge. The individual lines in each panel correspond to the value distributions under the nine discrete actions available to the agent. We emphasize that these plots were generated for the purpose of analyzing the learned models. In particular, the network only sees the current state when it outputs such distributions; it is not given with any information about the future.

Fig 3 clearly exhibits bi-modal distributions over values for non-survivors as much as 48 hours in advance of death. Further, as the patient gets closer to death, the mass shifts towards the left peak (which corresponds to death). This behavior is consistent with the patient’s deteriorating condition. Additionally, the distribution associated with the “no treatment” action has a larger left peak than others, highlighting that for these states the lack of treatment for even one hour can be fatal. The mass of the distributions for survivors, in contrast, is concentrated closer to the right limit *and* there is little difference between actions. Both of these observations are consistent with the expectation that survivors are less likely than non-survivors to enter the highest risk states, and so the consequences of a change in action/treatment are less extreme.

**Fig 4:**
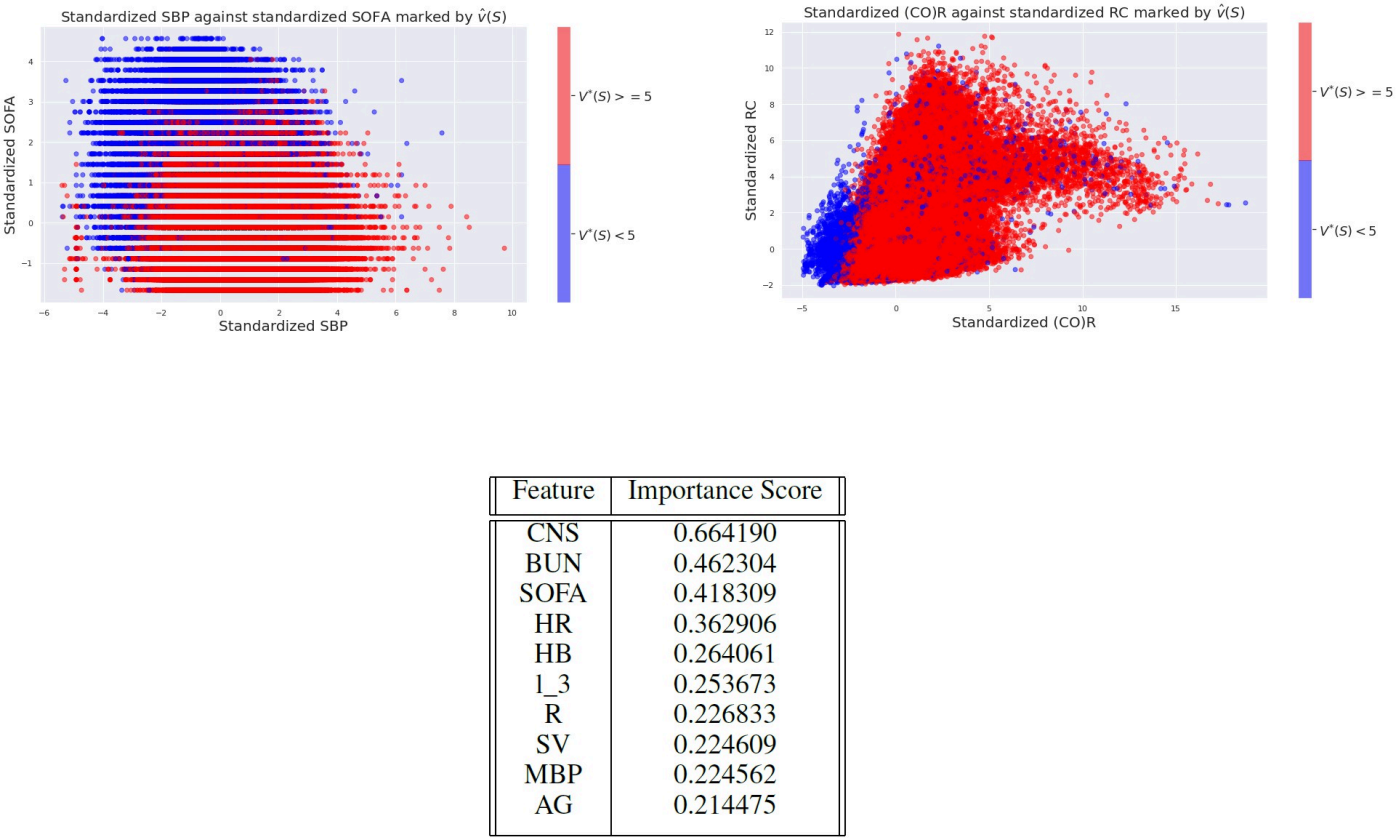
Scatter plots of scaled features: **Top row**: Marker colors indicates if ${\hat{V}}^{*}(S)<5$ (Blue) or ${\hat{V}}^{*}(S)\geq 5$ (Red) **Bottom**: Top 10 features measured by feature permutation. Here, *l*_*k* denotes the kth component of the latent lab representation.

We then investigated the dependence of features and inferred states on the value distributions and determined that they are explainable, and consistent with clinical expectation. For example, Fig 4 shows two scatter plots contrasting representative pairs of variables, stratified by an optimal expected value threshold of five. (This threshold was chosen arbitrarily, and we could observe similar results for any reasonable threshold.) It is clear that the model associates different states with different expected rewards/risk. For example, the model associates low SBP (hypotension) and high SOFA scores with an increased risk of death, which is consistent with medical knowledge. Thus the agent has learned to discriminate between low and high risk states in an explainable manner. The ability to learn such associations is noteworthy because the training and test data are highly imbalanced. In particular, 89% of states have the property V* ≥ 5.

Finally, we quantified the importance of each feature using feature permutation [28]. Briefly, for each patient we permute a selected feature while keeping others fixed. The mean absolute value difference of the *Q* function (across states and actions) is taken as the importance score for that patient. The above table lists the top 5 features across the entire cohort. The complete feature ranking can be found in the supplementary materials (Appendix C in S1 Text). The cardiovascular states and the latent lab representations are among the most important features, highlighting the importance of representation learning.

### 2.3 Vasopressor treatment strategies

**Fig 5:**
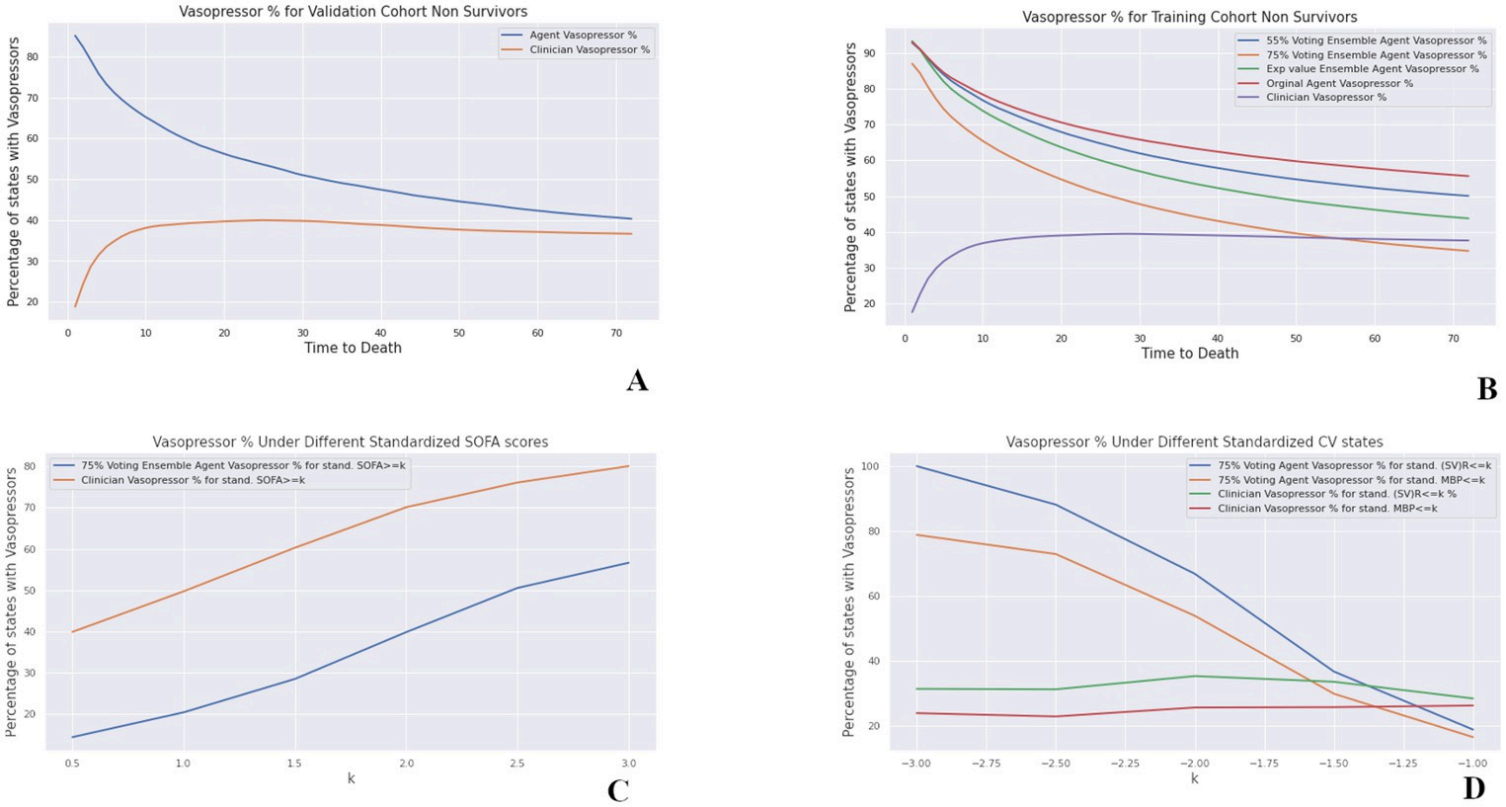
**Top row**: Percentage of states with vasopressors recommended for the training and validation states, with time to eventual death. Here a *p*% voting agent, denotes an agent which only prescribes vasopressors if an only if least *p*% of the Bootstrapped Ensembles have agree on giving vasopressors. **Bottom row**: The percentages of states with vasopressors recommended or given with respect to cardiovascular states and SOFA score.

We observed that the RL agents consistently recommend vasopressors for near-death (non-survivor) states, and that the percentage of such states increase closer to the patient’s eventual death. This phenomena is also shared by validation cohort states, as illustrated in Fig 5A, suggesting that this behavior isn’t due to overfitting. In contrast, clinicians have only administered vasopressors on average around 40% of the time, and this number drops off rapidly in the last 10 hours. We investigated whether these differences are an artifact of our choice of method by evaluating different training options and algorithms. Specifically, we: (i) trained networks with and without weighted experience sampling scheme (explained under Methods); (ii) used a different distributional RL algorithm, called Quartile Regression Q Learning [29]; (iii) considered an artificial voting ensemble agent, which only administers vasopressors if at least *p*% of the ensembles agree on giving vasopressors, at a given state; and (iv) considered the expected value of the ensemble agent, which takes a weighted average (weighted by the number of patients it’s trained on) of expected values of each bootstrapped network. In each case we observed similar results, as shown in Fig 5B.

We also investigated the relationship between vasopressor recommendation and cardiovascular states, and SOFA score. As illustrated in Fig 5D, the RL agents recommend vasopressors, much more regularly as (SV)R (product of stroke volume and resistance) and mean blood pressure drop. This is consistent with physiological knowledge, and latest critical care research. For example, [30] shows that hemodynamic effects of norepinephrine extends beyond blood pressure, and it effects SV and CO, and as described earlier, increasing systemic vascular resistance and blood pressure, are among the primary goals of vasopressor therapy. However, it is interesting to note that the clinicians have not necessarily associated lower blood pressure, or (SV)R with more frequent vasopressor administration. However they do seem to give vasopressors more regularly as SOFA score increase. These results could potentially provide an important direction and hints towards *better* treatment strategies.

This difference between the AI agent and human physicians is not unexpected, and does not imply that physicians are systematically acting sub-optimally. Rather, this difference reflects the fact that the rewards that the agents were trained on only consider the final state of the patient. They do not, for example, incorporate decisions that were made by the patient’s family to cease extraordinary measures, after consultation with the physician. Such status changes are common, but were not available in the training data.

In contrast to vasopressors, RL agents and clinicians had similar frequencies of fluid administration for non-survivors. However, there were some disagreement even amongst the ensembles on whether or not to administer fluids for survivors (at less risky states). We present a more detailed analysis along with global results in the supplementary information (Appendix C in S1 Text).

### 2.4 Uncertainty aware treatment

**Fig 6:**
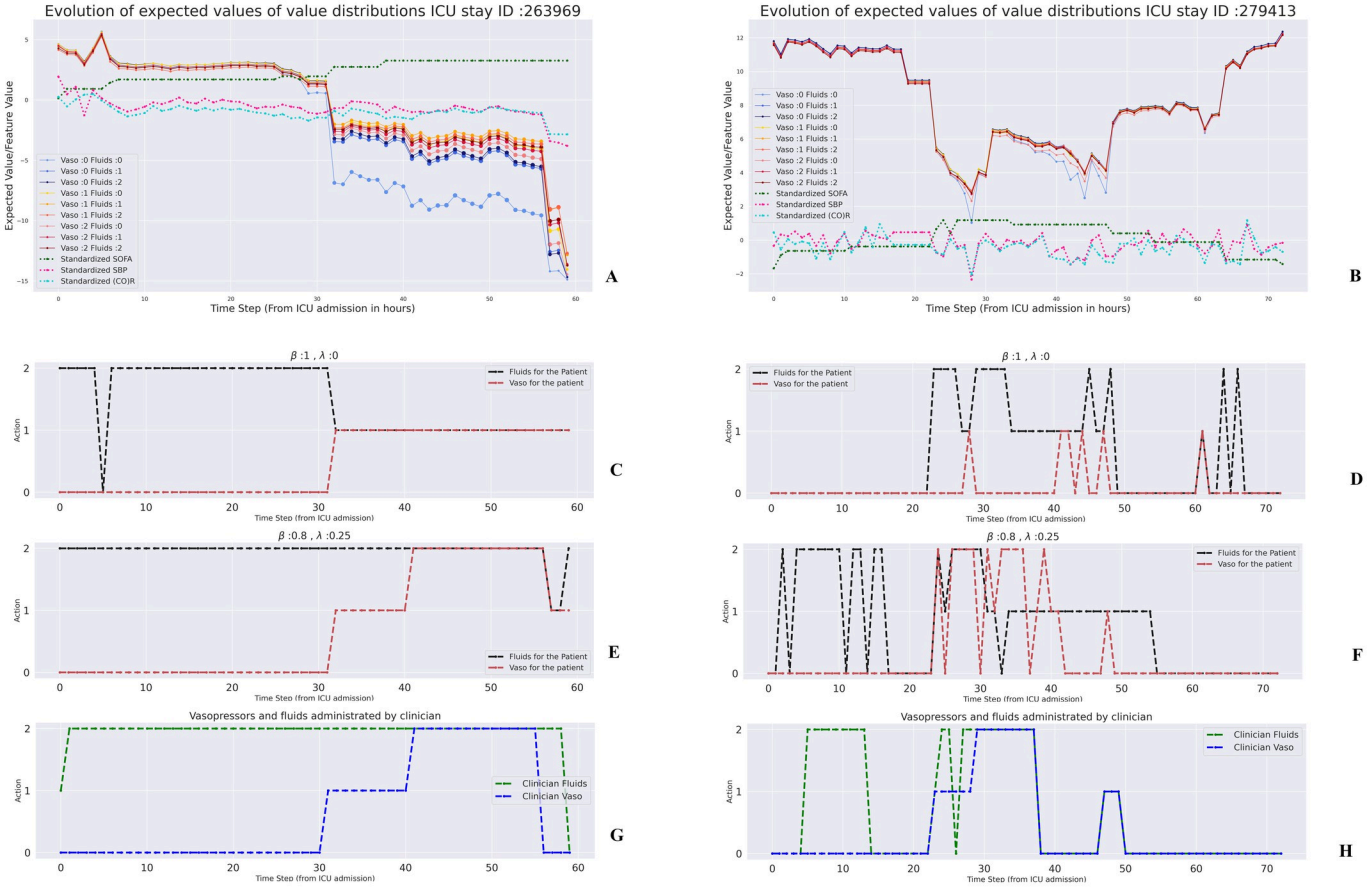
**Expected value evolution of the main agent for two patients: (A)** A patient who died in the ICU. **(B)** A survivor. The marker size indicates the parametric uncertainty associated with a particular action. Also shown are the standardized values of SOFA score, Systolic blood pressure, and the unidentifiable cardiovascular state (CO)R. The *x*-axis indicates the hours from ICU admission. **Recommended treatments under various preference parameters**: (see Eq 7). **(C)(E)** Recommendations for the same patient as in (A). **(D)(F)** Recommendations for the same patient as in (B). **Actual clinician treatments: (G)** treatment for the patient in (A), **(H)** treatment for the patient in (B).

Next, we consider representative patients, and analyze the expected values of all distributions and model uncertainty. Fig 6A shows the evolution of expected values for a non-survivor (ICU ID: 263969). This was typical among all non-survivors; initially there’s less variability among the expected values, but as the patient’s health deteriorates the variation becomes more drastic, and there is a clear preference towards vasopressor-based actions. The marker size indicates how much the agent is uncertain of its own results. We observe that the model is less certain when the patient’s health starts deteriorating. This can be attributed to the fact that these states are uncommon in the training data, and that the underlying cause driving deterioration can vary widely in septic patients.

For comparison, Fig 6B shows the expected values of a survivor (ICU ID: 279413). Here the expected values take a downward slide at around 25 hours from admission, with the values associated with no treatment considerably lower. This coincides with SOFA score increasing and SBP (CO)R rapidly decreasing, clearly indicating that the patient’s health is deteriorating. However, as SOFA score improves and the pressure and (CO)R goes up, the expected values do go up, and the difference between expected values of each distribution is considerably less. The uncertainty levels are also much lower.

The fact that expected values of different actions are close to each other in *healthy* patient states can be explained by Eq 2. State-action values are calculated under the assumption that the agent always takes the optimal action. Our agent chooses an action every hour, and the intermediate rewards are much smaller in value than the terminal rewards. Thus, the value of the choice of action is not likely to change very much in a *healthy* patient state from hour to hour. Put another way, any mistake made by the agent is easily reversed by taking the correct action in the next hour if the patient is non-critical. In contrast, in more critical states, a wrong action can have irreversible consequences.

Fig 6C–6F show different treatment recommendations under our proposed framework for uncertainty-aware decision support. Briefly, the user specifies their relative confidence in the RL-agent and a behavior cloner (which represents the human agent) by specifying a parameter, *β*. Lower values of *β* place more emphasis on the behavior cloner. An action preference score (see. Methods, Eq 7) is then calculated for each action in the current state. The score is a simple mixture of the scaled (using a softmax function) expected value of the ensembled distribution and the behavior probability, discounted by the model uncertainty corresponding to the state-action pair, using a parameter λ. Panels C-F illustrate that different choices are made, depending on the value of *β* and λ. Further, the sequence of treatments are qualitatively different for the non-survivor (panels C and E) and the survivor (panels D and F), because the agent has learned to identify critical states that require interventions; the average non-survivor tends to remain in such states for longer stretches, and so the agent makes relatively few adjustments, compared to the survivor. Once again, the agent does not know the ultimate fate of the patient. For comparison, panels G and H show the actual clinician treatments for the two patients.

### 2.5 Uncertainty quantification results

We now, briefly mention some interesting results on both model and environment uncertainties. Further results are available in the supplementary information (Appendix C in S1 Text).

**Fig 7:**
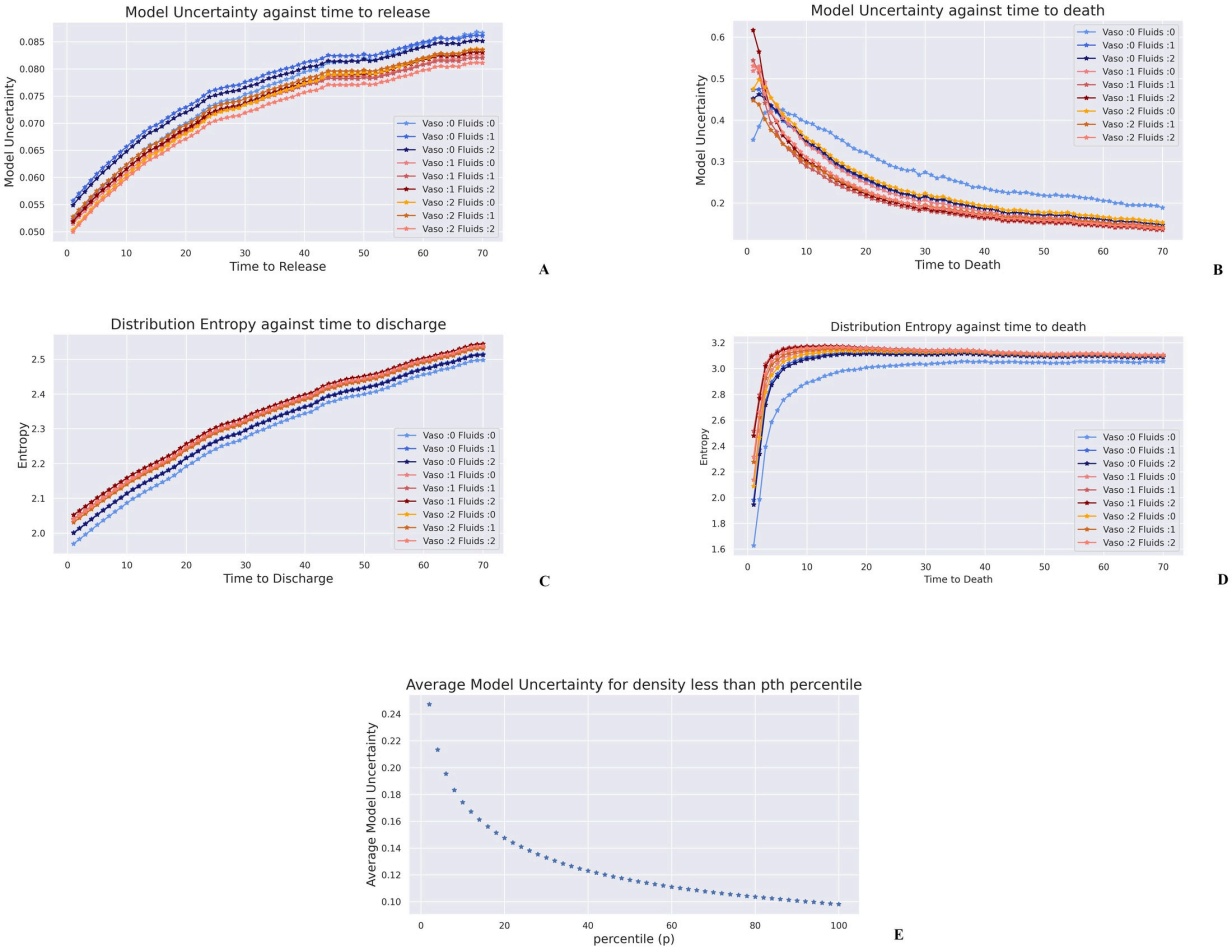
**(A)** Model Uncertainty with time to death for non-survivors, **(B)** Model Uncertainty with time to discharge for survivors **(C)** Averaged entropy of value distributions for non-survivors with time to death, **(D)** Averaged entropy of value distributions for survivors with time to release. **(E)** Average Model Uncertainty for data points with density less than the *p*-th percentile.

Fig 7A and 7B present how model uncertainty changes with time to death and release for non-survivors and survivors respectively. It is interesting to note that on average the model is a lot more uncertain about non survivors compared with survivors. Further, as a patient gets *closer* to death the uncertainty increases, whilst for survivors the model uncertainty decreases closer they are to ICU release. This observation is not surprising since death states are relatively uncommon, and also there are a wide variety of ways a septic patient may face increased mortality risk. However for survivors, we do expect all of them to approach a *healthy* state as they approach eventual discharge.

Fig 7C and 7D show the average entropy of the value distributions for each of the actions (again with time to death and release). This can be interpreted as a form of inherent environment uncertainty over future rewards. Now, there is less of a difference between the survivors and non-survivors and we can see a drastic drop in entropy for non-survivors as they approach death. This is not unexpected as the environment uncertainty should reduce when a patient’s state has deteriorated beyond a certain point. Similarly the entropy of value distributions reduce for survivors nearer they are to release. It is also interesting to note that on average vasopressor based actions have a lower model uncertainty but a higher entropy.

Next we fit a Gaussian Mixture model to the data, and examined the model uncertainty with the predicted likelihood. Fig 7E shows how the average (across all actions) model uncertainty for each data-point with a likelihood less than *p* th percentile. As one could expect the model uncertainty is higher for data-points with low density and reduces as the likelihood increases. This shows how the networks are uncertain of data away from the training distribution, and the value of having a large representative dataset.

### 2.6 A comment on off policy evaluation

Off policy evaluation (OPE) is the quantitative or statistical evaluation of the value of a learned policy, usually using another dataset. Although attractive in theory, most unbiased OPE methods use importance sampling, and are therefore dependent on a known behavior policy. This is not the case when the data were generated by human clinicians. Even if a suitable behavior policy *were* known, an obviously bad policy can result in a very high OPE value in our setting. For example, an agent that always prescribes no treatment for critical patients would, in effect, eliminate most of the rewards accumulated by non-survivors which are, of course, the source of the majority of the *negative* rewards. Such a policy would have a misleadingly high OPE, because human clinicians rarely withhold treatment for critical patients (the one exception being a conscious decision by the family to terminate extraordinary interventions), and so the the importance weights for such trajectories will tend towards 0.

We note that previous research has also argued at *all* OPE methods are unreliable in the context of sepsis management, and state-of-the-art OPE methods may fail to differentiate between obviously good and obviously bad policies [31]. However, we mention OPE results in the supplementary material (Appendix C in S1 Text). We do note that developing OPE techniques suited for the critical care domain is an important area of research to explore in the future.

## 3 Discussion and conclusion

We present an interdisciplinary approach which we believe takes a significant step towards improving the current state of data-driven interventions in the context of clinical sepsis, in terms of improving both outcome and interpretability. Indeed, we believe that the maximum benefit of Artificial Intelligence applied to medicine is best realized through the integration of mechanistic models of physiology whenever possible, uncertainty quantification, and human expert knowledge into sequential decision making frameworks.

Our contribution improves the status quo in several ways. Compared to prior work, our approach deals with partial observability of data, yet known physiology, by leveraging a low-order two-compartment Windkessel-type cardiovascular model in the context of self-supervised representation learning. As mentioned previously, this has several benefits. First, in the context of sepsis treatment, estimating the cardiovascular state is essential because the clinical decision to administer intravenous fluids or vasopressor is driven by an implicit differential diagnosis by the clinician, as to whether insufficient organ perfusion and shock are secondary to insufficient circulating volume (thus requiring fluids), vasoplegia (thus requiring vasopressors), or some combination of both fundamental pathophysiologies. Second, there is typically insufficient data to determine whether heart function is adequate (contractile dysfunction), but a mechanistic model provides an indirect means for estimating cardiac function by imposing known physiology. Finally, the incorporation of physiologic models improves model explainability, while deep neural networks and stochastic gradient-based optimizers make it possible to learn robust and generalizable representations from large data. We expect the unification of models based on first-principles and data-driven approaches will provide a powerful interface between traditional computational sciences and modern machine learning research, mutually benefiting both disciplines. We have not fully examined the association between inferred physiological state and treatment recommendation to confirm whether recommended actions are indeed clinically sensible. Such work is currently underway.

We also introduce an approach to quantifying model uncertainty, which is essential in any practical application of RL-based inference using clinical data. To the best of our knowledge, this is the first time uncertainty quantification is used to quantify epistemic uncertainty in RL-based optimization of sepsis treatment, and of critical care applications more generally. (Previous approaches ex. [11] have considered inherent environment uncertainty). The method’s uncertainty estimates, combined with the recommended action comprise a simple framework for automated clinical decision support. This principle aligns with the larger goal of combining different forms of expertise and knowledge for better decision making, a philosophy consistent with the rest of this work.

We chose a decision time step of one hour. Compared to similar work, this is much more compatible with the time scale of medical decision making in sepsis, where fluid and vasopressor treatments are titrated continuously. Accordingly, on such a time scale, there does not appear to be large differences in the relative merit of different dosing strategies. This makes intuitive sense: there is presumably a lesser need for major treatment modifications if decisions are made more frequently. Yet, a frequent finding across patients, especially the sickest ones, was that inaction (no intervention) was a consistently worse strategy. This also meets clinical intuition.

Reducing the time scale of decisions is not only appealing clinically in situation of rapidly evolving physiological states, such as is the case in early sepsis, but it also provides a more compelling basis for a less granular action space. Indeed, if decisions are made hourly, it does meet clinical intuition to have fever discrete actions. Few physicians will argue that there is likely to be little difference in administering 100cc or 200cc of fluids in the next hour. In the extreme, if time were continuous, the likely decision space at any given time, is whether a fluid bolus should be administered or not. A similar reasoning applies to vasopressors (increase, reduce, status quo). We further notice that our methods consistently identify high risk, non-survivor patient states which can *potentially* benefit from more frequent vasopressor treatment. These results should of course, be subject to clinical verification.

An important open problem in the application of offline RL to medicine is the means by which one evaluates learned treatment policies, given the obvious ethical issues associated with allowing an AI to exert some control over treatment. Still, proper clinical trials will be necessary, eventually, so the critical care community should define for itself the standards by which an AI would be deemed safe enough to enter clinical trials [32]. In this work, we have largely relied on a combination of medical expertise, and the fact that our model leverages prior knowledge in the form of a simple model of cardiovascular physiology, to argue that the learned policy is reasonable. We make no claim that the policy is expected to produce superior outcomes in sepsis patients, relative to human clinicians. One important area for future work may be the incorporation of more detailed models of physiology into our framework, or perhaps using such models in the context of *in silico* trials (ex. [33]) as a first step towards demonstrating that a learned policy is safe, and perhaps suitable for pre-clinical and clinical trials. Additional areas for future work include the design of alternative rewards (ex. based on time-dependent hazard ratios for death), and the application of risk-averse offline RL (ex. [34]).

## 4 Methods

### 4.1 Data sources and preprocessing

Our cohort consisted of adult patients (≥ 17) who satisfied the Sepsis 3 [35] criteria from the Multi-parameter Intelligent Monitoring in Intensive Care (MIMIC-III v1.4) database [36, 37]. We excluded patients with more than 25% missing values after creating hourly trajectories, and patients with no weight measurements recorded. The starting point of trajectories is ICU admission.

We further excluded patients who got discharged from the ICU but ended up dying a few days or weeks later at the hospital. Since we don’t have access to their patient data after the ICU release, treating the final ICU data as a terminal state would damage generalizability. We cannot treat those patients as survivors, however, as they were not released from the hospital.

Actions were selected by considering hourly total volume of fluids (adjusted for tonicity), and norepinephrine equivalent hourly dose (mcg/kg) for vasopressors. In computing the equivalent rates of each treatment, we followed the exact same queries as Komorowski et al [8]. When different fluids were administrated, we summed up the total fluid intake within the hour, and discretized the resulting distribution. For vasopressors, we considered the maximum norepinephrine equivalent rate administered within the hour to infer the hourly dose. We used 0.15 mcg/kg/min norepinephrine equivalent rate, and 500 ml for fluids, as the 1,2 cutoff when discretizing. These were chosen, considering the mean, median of non zero rates and medical knowledge, We also observe that due to the low dimensional action space, there is flexibility in choosing the cutoffs. A separate 0 action for each was added to denote no treatment.

Missing vitals and lab values were imputed using a last value carried forward scheme, as long as missingness remained less than 25% of values. A detailed description on extracting, cleaning and implementation specific processing as well as additional cohort details are included in the supplementary information (Appendix A and Appendix B in S1 Text).

### 4.2 Models

#### 4.2.1 Physiology-driven autoencoder

Autoencoders are a type of neural networks which learn a useful latent, typically lower-dimensional representation of input data, while assessing the fidelity of this representation by minimizing data reconstruction error. Our autoencoder architecture provides an implicit regularization by constraining the latent states to have physiological meaning, and the decoder to be a fixed physiologic model described in the next section. We further use a denoising scheme by randomly zeroing out input with a probability of 10–25%, when feeding into the network. This random corruption forces the network to take the whole patient trajectory (prior to the current time point) and previous treatment into account when producing its output, because it prevents the network from *memorizing* the current observation. In essence, we ask the inference network to predict observable blood pressures and the heart rate using corrupted versions of itself, by first projecting it into the cardiovascular latent state, and then decoding that to reconstruct.

More precisely, at time *t*, suppose the full history up to and including *t* is represented by *h*_*t*_. Then, the output of the system $\hat{o_{t}}$ satisfies,

$$\hat{o_{t}}=f\left(g\left(\tilde{h_{t}},a_{t},d\right)\right).$$

Here $\tilde{h_{t}}$ is the corrupted history computed as,

$$\tilde{h_{t}}=h_{t}\left(⊙\right)p$$

where *p* is a vector of same dimensions as *h*_*t*_ such that each element is sampled independently from a Bernoulli distribution, and (⊙) denotes element wise multiplication. *g*, *f* are the encoder and the decoder respectively, *a*_*t*_ denotes the treatment at time *t* and *d* denotes the demographic variables. The decoder *f* is detailed out in the next section, and *g* is the composition of neural networks as shown in Fig 1.

Fig 1C, shows the complete architecture of our inference network. As shown in the figure, the encoder is comprised of three neural networks, a patient encoder which computes initial hidden state estimates, a gated recurrent unit (GRU) [38] based recurrent neural network to encode the past history of vitals and scores up to and including the current time point, and a transition network which takes the previous state, the action and the history representation to output new cardiovascular state estimates. We train this structure end-to-end by minimizing the reconstruction loss, using stochastic gradient-based optimization. The supplementary material (Appendix B in S1 Text) provides a detailed description of model and architecture hyper-parameters, and training details.

**Cardiovascular model**. The cardiovascular model, is based on a two-element Windkessel model illustrated using the electrical analog in Fig 1B. This model provides a lumped representation of the resistive and elastic properties of the entire arterial circulation using just two elements, a resistance *R* and a capacitance *C*, which represent the systemic vascular resistance (SVR), and the elastance properties of the entire systemic circulation, respectively. Despite it’s simplicity, this model has been previously used to predict hemodynamic responses to vasopressors [39] and as an estimator of cardiac output and SVR [40].

The differential equation representing this model is:

$$\begin{array}{c} \frac{dP(t)}{dt}=-\frac{1}{RC}P\left(t\right)+\frac{Q(t)}{C} \end{array}$$ (3)

were *Q*(*t*) represents the volume of blood in the arterial system. As explained in [39], over the interval [0, *T*] (where *T* is the filling time of the arterial system) we can write *Q*(*t*) as *Q*(*t*) = *SVδ*(*t*), where *SV* stands for Stroke Volume, the volume of blood ejected from the heart in a heartbeat. When the system is integrated over the interval [0, *T*] we obtain the following expressions for *P*_*sys*_, *P*_*dias*_, *P*_*MAP*_, i.e., the systolic,diastolic, mean arterial pressure, respectively,

$$\begin{array}{c} P_{sys}=\frac{SV}{C}\frac{1}{1-e^{-T/RC}},\;P_{dias}=\frac{SV}{C}\frac{e^{-T/RC}}{1-e^{-T/RC}},\;P_{MAP}=\frac{(SV)R}{T}=\frac{(SV)FR}{60} \end{array}$$ (4)

*T* is the filling time and *F* is the heart rate, which is determined by *T*. This system of algebraic equations is used for the decoder of our autoencoder. Since heart rate can itself be affected by vasopressors and fluids, we added heart rate (*F*) as an additional cardiovascular state despite it being observable.

Therefore we have a multivariate function *f*: {*R*, *C*, *SV*, *F*, *T*} → {*P*_*sys*_, *P*_*dias*_, *P*_*MAP*_, *F*}, represented by the equations above, and the trivial relationship *F* = *F* (Despite the obvious relationship we used both *F* and *T*, for ease of training and stability.) As stated previously, to prevent it from just using the current observations, we use a denoising scheme for training. This ensures at a fixed time, the model cannot *memorize* the current observation and learn to invert *f*, since there is a nonzero probability of corruption. Thus it has to learn to factor in the history and the treatments when determining the cardiovascular states. Once *SV* is inferred, the cardiac output (CO), can be computed as *CO* = (*SV*)*F*.

Since *f* is not one to one, typically not all states are identifiable. To arrive at a better approximation we used the latent space to only model deviations from fixed baselines. We also posit that identifiable combinations of states, when trained with a denoising scheme, should provide important cardiovascular representations in the POMDP setting.

#### 4.2.2 Denoising GRU autoencoder for representing Lab history

We use another recurrent autoencoder to represent patient lab history, motivated by the fact that labs are recorded only once every 12 hours. Forward filling the same observation for 12 time points, is almost certainly sub-optimal, and the patterns of change in lab history can be helpful in learning a more faithful representation. Thus, we use a denoising GRU autoencoder constructed by stacking three multi-layer GRU networks on top of each other, with a decreasing number of nodes in each layer, the last 10 dimensional hidden layer was used as our representation. This architecture is motivated by architectures used in speech recognition [41].

This model was also trained by corrupting the input, where each data-point was zeroed with a probability of up to 50%. (The rate was gradually increased from 0 to 50%). As with the previous autoencoder, this provides an extra form of regularization, and forces the learned representation to encode the entire history.

Model architecture and training details and presented in the supplementary materials (Appendix B in S1 Text).

#### 4.2.3 Behavior cloner

We use a standard multi-layer neural network as our imitation learner. This model is trained using stochastic gradient-based optimization by minimizing the negative log-likelihood loss, between the predicted action and the observed clinician action, with added regularization to prevent overfitting.

We do mention that there are many other options that could be used as a imitation learner, including nearest neighbor-based method as in [10].

### 4.3 POMDP formulation

**States**. A state is represented by 41 dimensional real-valued vector consisting of:

- **Demographics**: Age, Gender, Weight.
- **Vitals**: Heart Rate, Systolic Blood Pressure, Diastolic Blood Pressure, Mean Arterial Blood Pressure, Temperature, SpO2, Respiratory Rate.
- **Scores**: 24 hour based scores of, SOFA, Liver, Renal, CNS, Cardiovascular
- **Labs**: Anion Gap, Bicarbonate, Creatinine, Chloride, Glucose, Hematocrit, Hemoglobin, Platelet, Potassium, Sodium, BUN, WBC.
- **Latent States**: Cardiovascular states and 10 dimensional lab history representation.

**Actions**. To ensure each action has a considerable representation in the dataset, we discretize vasopressor and fluid administrations into 3 bins, instead of 5 as in previous work [9], [8] [10]. This results in 9 dimensional action space.

**Timestep**. 1 hour.

**Rewards**. We use the reward structure that was suggested by Raghu et. al [9], with a minor modification. Since lactate was very sparse amongst out cohort we only considered SOFA based intermediate rewards. Specifically, whenever *s*_*t*+1_ is not terminal, we use reward of the form:

$$\begin{array}{c} r\left(s_{t},a,s_{t+1}\right)=-0.025I(\left(s_{t+1}^{SOFA}=s_{t}^{SOFA}\;\&\;s_{t+1}^{SOFA}>0\right)-0.125I(s_{t+1}^{SOFA}-s_{t}^{SOFA}), \end{array}$$ (5)

For terminal rewards we put *r*(*s*_*t*_, *a*, *s*_*t*+1_) = 15 for survival and *r*(*s*_*t*_, *a*, *s*_*t*+1_) = −15 for non-survival.

#### 4.3.1 Training

We only mention important details of training the RL algorithms here. Representation Learning related training and implementations are detailed out in the supplementary information (Appendix B in S1 Text).

We train the Q networks using a weighted random sampling-based experience replay, analogous to the prioritized experienced replay [42], which has resulted in superior performance in classical DRL domains, such as Atari games.

In particular for each batch, we sample our transitions from a multinomial distribution, with higher weights given to terminal death states, near death states (measured by time of eventual death), and terminal surviving states. We used a batch size of 100, and adjusted weights such that on average there is 1 surviving state, and 1 death state in each batch.

This does introduce bias, with respect to the existing transition dataset, however we argue that this would correspond to sampling transitions from a different data distribution, which is closer to the true patient transition distribution, we are interested in, as we are necessarily interested in reducing mortality. We empirically observe that, when using such a weighting scheme the value distributions align more closely to clinical knowledge in identifying risky states, and *near* death states.

A same weighting scheme was used for all ensemble networks, which are trained to estimate uncertainty. As mentioned previously, we verified that the main results on vasopressor treatment strategies hold even for pure random sampling.

### 4.4 Uncertainty

In this section, we consider model uncertainty, and not the inherent environment uncertainty. Model uncertainty stems from the data used in training, neural network architectures, training algorithms, and the training process itself.

Inspired by statistical learning theory [43], and the associated structured risk minimization problem [44], we define the model uncertainty, (conditioned on a state *s* and a action *a*), given our learning algorithm, and model architecture as:

$$\begin{array}{c} E_{\theta ,D}\left[l\left(\theta ,E_{D}\left[\theta \right]\right)|s,a\right]=\int l\left(\theta ,E_{D}\left[\theta \right]\right){{|}_{s,a}p\left(\theta ,D\right)d\theta dD=\int l\left(\theta ,E_{D}\left[\theta \right]\right)|}_{s,a}p\left(\theta |D\right)p\left(D\right)d\theta dD \end{array}$$ (6)

Here, *D* denotes the unknown distribution of ICU patient transitions that we are attempting to learn our policies with respect to. *θ* is a random variable which characterizes the value distributions. (For the C51 algorithm this can be interpreted as an element in $R^{51}$). This is outputted by our networks trained on a dataset sampled from *D*, for a given state action pair. This random variable is certainly dependent on the training data, and the randomness stems from the inherent randomness of stochastic gradient based optimization [45] and random weights initialization. The quantity *l* is a divergence metric appropriate for comparing probability distributions. We use the Kullback–Leibler divergence [46] for *l*.

#### 4.4.1 Estimating the uncertainty measure

We construct a Monte-Carlo estimate of the integral in (6) by bootstrapping 25 different datasets, each substantially smaller than the full training dataset, and training identical distributional RL algorithms in each. This can be done efficiently due to the sample efficiency of distributional methods. Additionally, we can approximate E[θ] either by the ensemble value distribution, or by the value distribution of the model trained on the full training dataset.

### 4.5 Uncertainty aware treatment

In this section, we describe a general framework for choosing actions that factors in uncertainty. Notice that, because our RL algorithm learns (an approximation of) the optimal value distributions, making decisions by considering additional information does not violate any assumption underlying the learning process.

When suggesting safe treatment strategies, we want the proposed action to have high expected value, however we would also like our agent flexible enough to propose an action with less model uncertainty, if two actions have very close expected values to each other. Another important factor to consider is how likely an action is to be taken by a human clinician. This will have significance in a situation where human expertise is scarce. Large retrospective datasets subsume experience of hundreds of clinicians, and knowing what previous clinicians have done in similar situations, will be valuable such situations. Therefore we use behavior cloning to learn an approximate behavior policy of clinicians on average.

To satisfy all three goals, we propose a general framework for choosing actions, based on an action preference score, P(s,a), parameterized by two parameters. This general framework is flexible, yet simple, and the end-user can choose the parameters to reflect their own expert knowledge, and confidence of the framework.

Let *G*(*s*, *a*) be a human behavior likelihood score function. In this work we equate *G*(*s*, *a*) with the probabilities outputted by the behavior cloning network described in section 6.2.3. Given a state *s*, we define P(s,a) associated with each action *a*, as:

$$\begin{array}{c} P\left(s,a\right)=\beta (Softmax\left(\tilde{Q^{*}}\left(s,a\right)\right)+\left(1-\beta \right)G\left(s,a\right)-\lambda u\left(s,a\right) \end{array}$$ (7)

where *β*, λ ≥ 0, *u*(*s*, *a*) is the parametric uncertainty associated with the state-action pair, *s*, *a*, *G*(*s*, *a*) is the behavior likelihood probability and $\tilde{Q^{*}}\left(s,a\right)$ is the *Q* function computed from the ensembled value distributions. When human expertise is available, *G*(*s*, *a*) can be modified or even re-defined to factor in expert opinion. λ penalizes uncertainty, and a low *β* forces the action to be close to a clinician action. We could recover the expected value criteria by setting *β* = 1, λ = 0, and we could use the system as a pure behavior cloner, by setting *β* = 0, λ = 0. Therefore *β* controls how far from the highest expected value/behavior likelihood score can the agent choose an action.

## Supporting information

S1 Text In this section, we provide brief descriptions of the supplementary text file, S1 text. This provides additional exposition on results and methods complimenting the main text and is divided into 4 sections. **Cohort Details (Appendix A)**: This appendix summarizes our cohort presenting summary statistics on the patients. **Neural Network Architectures and Implementation Details (Appendix B)**: This section presents a detailed description of the neural networks used, implementation details and hyper-parameters involved. These include the representation learning, RL and uncertainty quantification. **Additional Results (Appendix C)**: This section presents further results. We do this in three sub sections. **RL Results**: We present additional results of Reinforcement Learning. This section also include figures showing feature importance scores for all the features, heat maps presenting the recommended actions under different schemes, and expected value evolution for validation patients. **Uncertainty Quantification Results**: We continue our discussion on uncertainty quantification results. The section presents a table which shows the mean model uncertainties for non survivors and survivors, stratified by cohort and the action. **OPE Results**: This subsection includes results from OPE. The results include value estimates under different preference scores and cover all the ensembles. However, these results are subject to the caveats mentioned previously. **Limitations and Open Problems (Appendix D)**: We conclude with a high-level discussion of limitations of both our and general RL methods. We further detail out some ideas for future work.
(PDF)
