# Supplementary material for: Unifying cardiovascular modelling with deep reinforcement learning for uncertainty aware control of sepsis treatment
Source: PLOS Digit Health. 2022 Feb 17;1(2):e0000012. doi: 10.1371/journal.pdig.0000012 (PMC9931225; doi:10.1371/journal.pdig.0000012)
Supplement: S1 Text — In this section, we provide brief descriptions of the supplementary text file, S1 text. This provides additional exposition on results and methods complimenting the main text and is divided into 4 sections. Cohort Details (Appendix A): This appendix summarizes our cohort presenting summary statistics on the patients. Neural Network Architectures and Implementation Details (Appendix B): This section presents a detailed description of the neural networks used, implementation details and hyper-parameters involved. These include the representation learning, RL and uncertainty quantification. Additional Results (Appendix C): This section presents further results. We do this in three sub sections. RL Results: We present additional results of Reinforcement Learning. This section also include figures showing feature importance scores for all the features, heat maps presenting the recommended actions under different schemes, and expected value evolution for validation patients. Uncertainty Quantification Results: We continue our discussion on uncertainty quantification results. The section presents a table which shows the mean model uncertainties for non survivors and survivors, stratified by cohort and the action. OPE Results: This subsection includes results from OPE. The results include value estimates under different preference scores and cover all the ensembles. However, these results are subject to the caveats mentioned previously. Limitations and Open Problems (Appendix D): We conclude with a high-level discussion of limitations of both our and general RL methods. We further detail out some ideas for future work. (PDF) [file pdig.0000012.s001.pdf]

Supplementary Information

Appendix A: Cohort Details

Our total patient cohort consists of 18,472 patients, out of which 1,828 were non-survivors.

Table 2: Cohort Details

Cohort	% Female	Mean Age	Mean ICU Stay	Total Population
Overall	42.33 %	66.05	7 days 15 hours	18472
Non-Survivors	42.67 %	68.8	9 days 13 hours	1828
Survivors	42.14 %	65.91	5 days 13 hours	16644

This resulted in an experience replay consisting a total of 2596604 transitions.

Appendix B: Neural Network Architectures and Implementation Details

Physiology-driven Autoendcoder

Encoder :

- Patient Encoder: Multi-layer feed-forward neural network, with 3 hidden layers with 64 nodes each, followed by exponential linear unit, (eLU) non-linearity applied element wise.
- Transition: Multi-layer feed-forward neural network, with 8 hidden layers with 128 nodes each, followed by exponential linear unit, (eLU) non-linearity applied element wise.
- RNN: Gated recurrent unit, based RNN, with 1 hidden layer, with 64 nodes.

We note that, as inputs for the network specifically the RNN, we included all vitals, and SOFA-related scores, including the four dimensional observations, systolic blood pressure, diastolic blood pressure, mean blood pressure and heart rate.

For training, we used Adam [45], with a low learning rate (1e-5), the corruption was only introduced after the model has been trained for several epochs. For RL representation we used the model trained with 10% corruption.

Denoising Lab Autoencoder

This is comprised of three GRU networks stacked on top of each other.

- Network 1 : 12 hidden units, with 512 nodes, outputs a 128 node vector.
- Network 2 : 5 hidden units 128 nodes each, outputs a 10 dimensional vector.
- Network 3: 3 hidden units, with 10 nodes each, the last of which is taken as our hidden lab representation.

We train this again using Adam, and corruption is gradually introduced starting from 0% to 50%. We use the network trained under 50% corrupted inputs, when inferring the hidden lab representation for RL.

We standardized all the labs before feeding into the network.

Imitation Learning

Muli-layer neural network with 4 hidden layers: 3 with node size 512, and the last 256. All hidden (and input) layers are followed by a rectified linear unit (reLU) non-linearity.

Training was again using Adam with a standard learning rate, and we minimized a negative log-likelihood loss, which is standard in classification problems.

Bootstrapping and Deep ensembles

To learn each bootstrapped network, we first sampled from the all patients to arrive at a bootstrapped patient list. Then we train the networks, for 2 or 3 epochs each (to have further randomness), using a process identical to training the main RL algorithm.

For the uncertainty quantification step, we trained the majority of bootstrapped ensembles on as little as 40% of total patients, however for the results presented under vasopressor administration, we only considered ensembles which were trained on a cohort of 65-80% of patients. The number of patients was also picked at random. There were 20 such bootstrapped ensembles.

Distributional Q learning

We use the standard C51 training algorithm as in [23]. Q network was a multi-layer neural network. Apart from the weighted sampling described in the main body of the text, training steps were all standard.

We use a target network, and update the target networks using polyak target updating with $\tau = 0.005$. (i.e. after every iteration/training step we set the target network weights to a linear combination of it's own weights, weighted by (1-τ) an the Q network weights, weighted by τ). This kind of target network is common amongst all deep Q learning, algorithms.

We summarize the hyper-parameters involved in table below.

Table 3: RL algorithm hyper-parameters

Hyper-parameter	Value
Support size	51
Maximum value	18
Minimum value	-18
γ	0.999
Batch size	100
Number of iterations	51932
Optimizer	Adam
Learning rate	$3 * 10^{-4}$
τ	0.005

Appendix C: Additional Results

RL Results

In this section, we present further results of the distributional RL algorithm, and uncertainty quantification. First, in Fig 8 we present the feature importance of all features.

Fig 9 shows, expected value trajectories, of randomly selected validation patients. As we have mentioned previously these results indicate the generalizability of our value networks.

To be consistent with previous work (e.x. [9]), we present heat plots of global actions, overall, low SOFA (< 5), medium SOFA ($\geq 5 < 15$) and high SOFA (> 15) separately. We further provide last 24 hours on non-survivors and results from decisions taken with respect to the expected value of the an ensembled weighted distribution, (corresponding to $\beta = 1, \lambda = 0$) and $\beta = 0.8, \lambda = 0.25$.

However, at its core, our approach strives to extract and use patient specific recurrent representations to learn personalized treatments. Therefore global analysis is unlikely to provide much insight into the intricacies that underline the decision making process. Further when analyzing the proposed treatment, it should be noted that each action is proposed considering only the current, actual state. Therefore for a fixed patient trajectory at a fixed time, the agent does not know what it has proposed previously, nor how its action would have impacted the state.

The most striking difference is for non survivors near death states. Our methods consistently recommend vasopressors. It is also interesting that RL methods have in general preferred low/medium (corresponding to 1) vasopressors and fluids as opposed to high doses (2). Just as we mentioned in the main text, when ensembled, agents do not recommend fluids for survivors' less critical states. It must be noted however that the agent trained on the whole cohort did have fluids recommended regularly, but there is disagreement amongst the ensembles.

Uncertainty Quantification Results

In this section, we briefly mention results of uncertainty quantification.

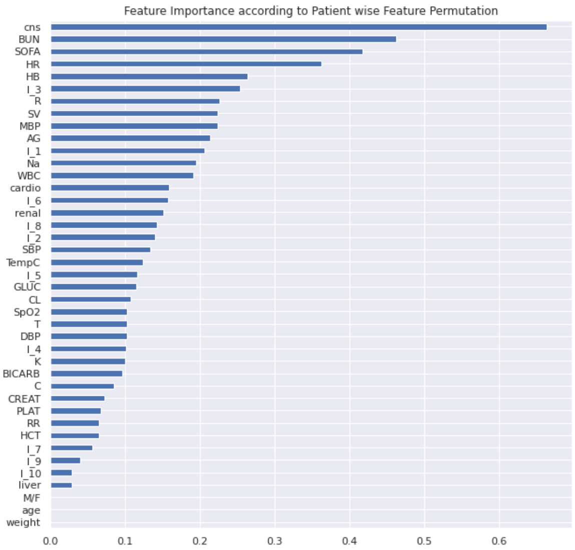

Fig. 8: Feature Importance measured by feature permutation

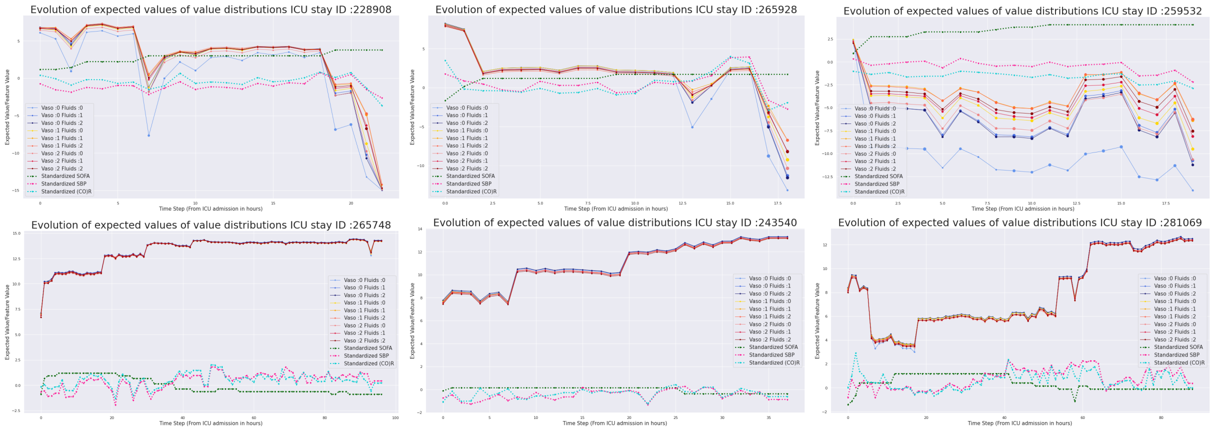

Fig. 9: Expected Values of random validation patients, **Top**: Non-survivors, **Bottom**: Survivors. As with Fig 4, the blob size indicate the uncertainty

The common pattern is that for most patients who have died, the model is less confident about its value distributions as they become closer to death. Uncertainty among each action varies from patient to patient. However for survivors this behavior is the exact opposite, as the agent is more confident of its results and becomes even more confident as the patient gets closer to discharge from the ICU. We illustrated this in Fig 7 in the main text, which presented averaged model uncertainty with time to death and discharge for non-survivors and survivors respectively.

Table 4 presents average uncertainty, among all patient states, grouped by the training and validation datasets and whether the patient was a survivor or a non-survivor. As we mentioned in the main text, the uncertainty is much higher for non survivors than survivors. Further uncertainties for validation non-survivors are higher than training non-survivors. However for survivors the training and validation uncertainty are very similar on average.

Both Table 4 and Fig 7 agree with our expectations, because near-death states, are relatively uncommon, and also there could be a lot of different ways a septic patient may have increased mortality risk. However, for survivors, we do expect our agent to be confident of their survival, as their states should approach a *healthy* state.

OPE Results

Despite the inherent limitations of OPE methods, we present results of Weighted Importance Sampling (WIS) OPE estimates of the validation cohort. Here, we compute the OPE estimates assuming our agent takes an action based on a score (either the expected value or the preference score in Equation 7) with a probability of 0.99 and takes a random

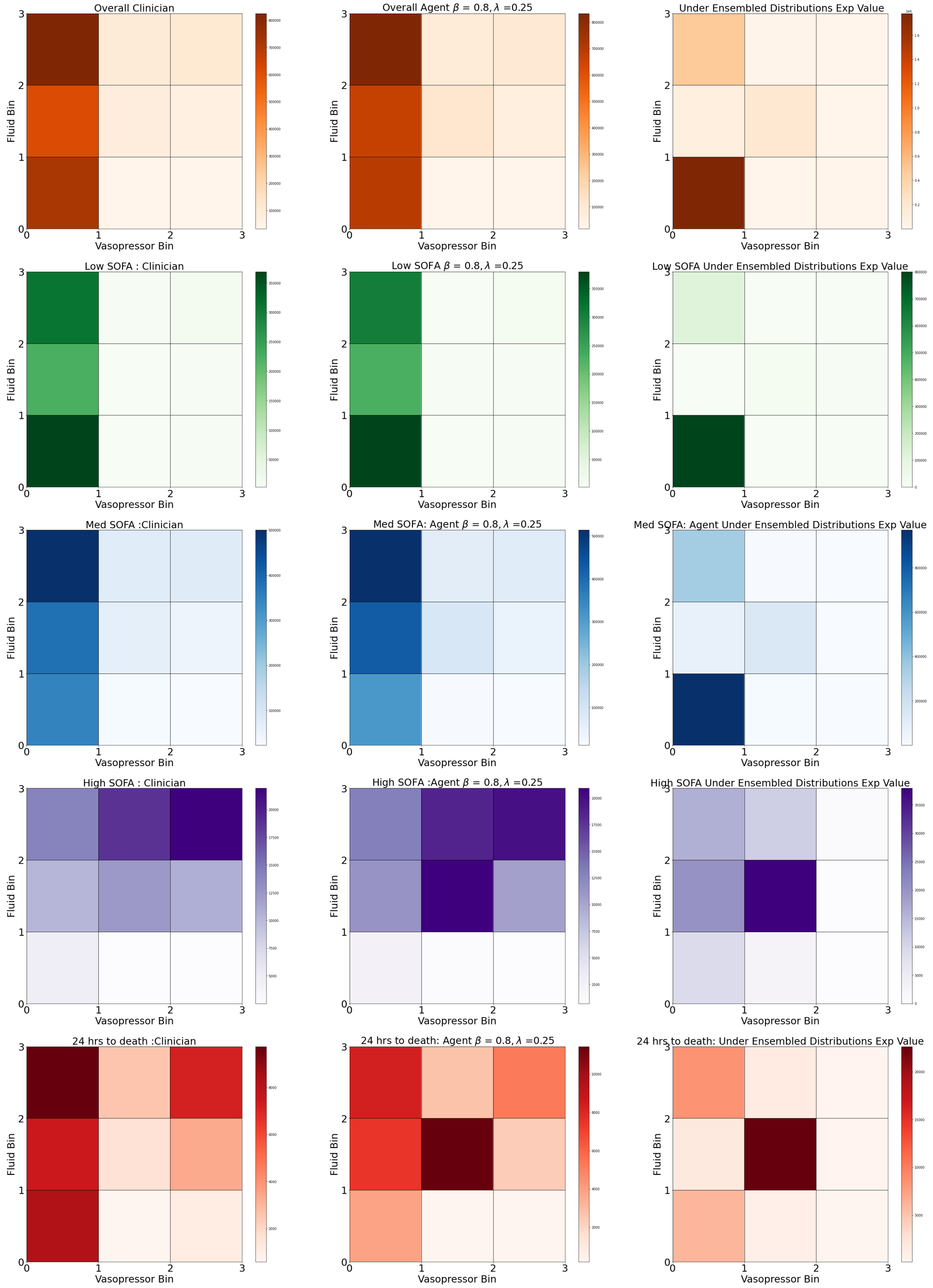

Fig. 10: **L** : Heat-plots for recommended actions, under $\beta = 0.8$, $l\lambda = 0.25$ and Ensembled Distribution Expected Values. Shown are clinician's vs Agent for overall (orange), low sofa (green), medium sofa (blue), high score (purple) and non survivors last 24 hrs (red).

Table 4: Mean Model Uncertainty for Survivors and Non-Survivors in training and validation datasets

Action/Cohort	Non-Survivors Train.	Survivors Train.	Non Survivors Val.	Survivors Val.
Vaso 0 Fluids 0	0.1916	0.0887	0.3617	0.0861
Vaso 0 Fluids 1	0.1591	0.0855	0.3085	0.0894
Vaso 0 Fluids 2	0.1587	0.0846	0.3104	0.0879
Vaso 1 Fluids 0	0.1547	0.0820	0.3066	0.0850
Vaso 1 Fluids 1	0.1451	0.0815	0.2676	0.0847
Vaso 1 Fluids 2	0.1482	0.0827	0.2776	0.0850
Vaso 1 Fluids 0	0.1634	0.0839	0.3135	0.0853
Vaso 2 Fluids 1	0.1498	0.0831	0.2710	0.0860
Vaso 2 Fluids 2	0.1488	0.0808	0.2850	0.0832

action with a probability of 0.01. This was done to make the policy stochastic, because taking importance sample based estimates of deterministic policies can be problematic.

For a dataset D the WIS value estimate is computed as,

$$WIS(D) = \frac{1}{\sum_{i=1}^{n} w_i} \sum_{i=1}^{n} w_i (\sum_{t=1}^{L^i} \gamma^t (r_t^i)) \tag{8}$$

Where $w_i = \prod_{t=0}^{L^i} \pi_e(a_t|s_t)/\pi_b(a_t|s_t)$, a_i is the action taken in the dataset, π_e is the policy being valuated and π_b is the behavior policy. And L^i is the length of the trajectory of the ith patient

As mentioned in the main text, Importance Sampling based OPE methods require a known behavior policy. We estimate this by training a neural network as a behavior-cloner on the observable variables.

The clinicians' value estimate for the validation cohort was 12.44. The OPE value estimate for the ensemble agent taking actions with $\beta = 0.8, \lambda = 0.25$ was 13.03. The ensemble agent taking actions under expected value resulted in an OPE value estimate of 13.3. We further evaluated the value estimates on each of the bootstrapped ensemble. These numbers are shown as a box plot in Fig 11. Whilst all of the values were greater than the clinicians' value, for the reasons explained in the main text we note that these results don't necessarily imply that the RL agent is superior to the clinicians.

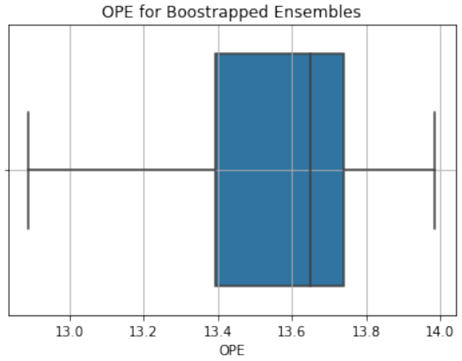

Fig. 11: OPE value estimates for bootstrapped ensembles

Appendix D: Limitations and Open Problems

As stated in the main text, and discussed in previous work, the main limitation of *any* data driven or computational approach to finding optimal treatment is proper evaluation of the learned policy. In this work we relied on medical expertise and physiologic knowledge in interpreting the results, but evaluating learned policies is an active research area in offline RL, and future research could find better methods which are more suited to critical care applications.

A related issue is model selection. Like supervised learning, it has been shown previously that training deep RL algorithms longer on the same dataset can result in poor performance and overfitting. A lack of an obvious evaluation metric (such as test accuracy for a classification problem) makes model selection complicated. We used results after only two full passes of the dataset (51932 iterations), observing that the results don't make the same sense, clinically, when it is trained for too long. Indeed Fig 12 shows the expected value evolution for a validation cohort non-survivor for different weights. As we can see, its results are far too optimistic when the patient is a few hours away from death, if trained longer. However, the vasopressor recommendation results for non-survivors, which was presented earlier, do hold for all the different training weights.

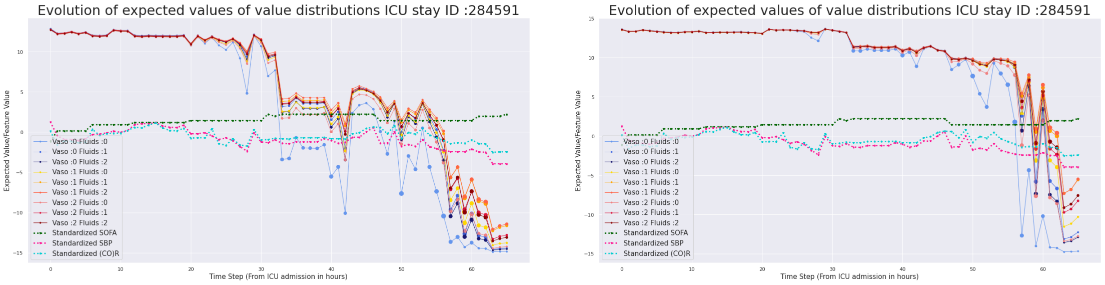

Fig. 12: Expected Values of non-survivor, **Left**: Trained for 2 epochs, **Right** : Trained for 7 epochs

In the context of sepsis treatment, as we mentioned under Discussion, a further challenge is designing rewards. For example even *survivors* have a high risk of relapse, and their physiologic age is significantly higher than their actual age. Therefore it can be argued that survival at the ICU should not be *rewarded* as much as (in absolute value) death. Further organ damage and mortality could be competing objectives for some patients. Whilst it is possible to have a weighted combination, of both as we did, a multi-objective RL framework could also be looked into. As we mentioned before we hope to explore these questions in future work.

4.5.1 Future Work

There are other avenues we would like to explore.

Model-based RL with physiological models: Model-based RL aims to explicitly model the underlying environment and then use this information in various ways for control. [8] This paradigm provides a natural place to incorporate mechanistic models, which could potentially help both control and interpretability. Clearly, the availability of more granular data, or of additional domains of data, could allow better estimation of the underlying physiological model and thus reduce uncertainty.

Reward Structure: Our reward structure was based on previous work and has clinical appeal. However, rewards are an essential component of any RL algorithm and is the only place where the agent can judge the merit of its proposed actions. This is potentially another place to include physiological knowledge. Ideally, we would want our reward structure to capture an accurate mortality risk, and an organ damage score, with each state. Risk-based rewards, rooted in anticipated evolution over a meaningful clinical horizon, should be considered in future schemes.

Risk Averse RL: It could be argued that, maximizing the sum of expected future rewards may not best reflect the end goals of safety critical domains. Whist the rewards can be engineered to promote risk aversion, risk averse RL is a fast growing research area, which we are keen to explore, if the RL objective itself can be tweaked to be more suitable for critical care research.

[8] It could in theory be argued that our work itself is a model based and model free hybrid method.
